# The effect of the 2-week wait referral system on the detection of and mortality from colorectal cancer: protocol of a systematic review and meta-analysis

**DOI:** 10.1186/s13643-016-0358-6

**Published:** 2016-10-26

**Authors:** Ella Mozdiak, Alexander Tsertsvadze, Michael McFarlane, Monika Widlak, Maria Tabuso, Amber Dunlop, Ramesh Arasaradnam

**Affiliations:** 1 Department of Gastroenterology, University Hospitals Coventry and Warwickshire, Clifford Bridge Road, Coventry, CV2 2DX UK; 2 Warwick Medical School, University of Warwick, Coventry, CV4 7AL UK; 3 Communicable Disease Control Epidemiology and Evidence, Populations, Evidence and Technologies, Division of Health Sciences, Warwick Medical School, University of Warwick, Coventry, CV4 7AL UK; 4 University Hospitals Coventry and Warwickshire, Clifford Bridge Road, Coventry, CV2 2DX UK

## Abstract

**Background:**

Colorectal cancer represents the fourth most common cancer in England and Wales; survival is high for early stage disease but declines sharply with advanced stage. UK figures suggest that cancer survival rates are lower than those of other Western European countries. Current 5-year survival is around 50 %. A rapid access strategy was introduced through the Department of Health in 2000. This 2-week wait (TWW) referral pathway was devised to streamline referral for suspected cancer, allow diagnosis at an earlier stage, reduce cancer survival inequality and reduce cancer-related mortality. However, only around half of patients with colorectal cancer have symptoms that fit the TWW criteria plus there is a fourfold difference in referral rates across England and Wales.

High-quality evidence of TWW outcome measures for colorectal cancer is lacking. This systematic review will collate and evaluate the latest evidence on colorectal cancer detection rate, stage at diagnosis and mortality.

**Methods:**

English-language publications from 2000 reporting outcomes on the TWW referral system for suspected colorectal cancer will be eligible for inclusion. Cochrane, EMBASE, MEDLINE via PubMed, NHS Evidence, Trip and the British Library Catalogue databases will be searched. Two paired reviewers will independently screen all titles/abstracts and full text for eligibility, then extract data and assess for bias using standardised formats. They will hand review reference lists of eligible articles. Disagreement will be resolved via third party adjudication. Summary effect measures for post-referral diagnosis and mortality rates will be calculated and expressed as relative risk, hazard rate ratio or risk difference with corresponding 95 % confidence intervals. Where possible summary effect measures will be pooled, heterogeneity and its extent for pooled estimates will be assessed via visual inspection of forest plots and explored via sub-group analysis.

**Discussion:**

In this systematic review, we aim to summarise the relevant evidence on cancer detection rate, cancer stage at diagnosis and disease-related mortality rates for patients with suspected colorectal cancer investigated through the TWW referral system in England and Wales. We will highlight gaps in the evidence and provide a better understanding of whether it is meeting its desired effect.

**Systematic review registration:**

PROSPERO CRD42016037368

**Electronic supplementary material:**

The online version of this article (doi:10.1186/s13643-016-0358-6) contains supplementary material, which is available to authorized users.

## Introduction

### Health and economic impact

Colorectal cancer represents the fourth most common cancer in England and Wales and is the second leading cause of cancer-related deaths [1]. It makes up over 10 % of all cancer diagnoses in the England and Wales population, and like many cancers, it predominantly affects older age groups, especially people aged 60 years or older. The financial burden associated with colorectal cancer is significant. It is estimated that total colorectal cancer cost amounts up to £1.6 billion per year in the UK; this includes the economic, healthcare and the unpaid care costs provided by family and friends [2].

The diagnosis of colorectal cancer can be difficult as symptoms are variable, with many patients reporting no symptoms at all, especially at early stages of the disease. The gold standard of diagnosis is colonoscopy, which enables tissue biopsy. However, CT colonography is being increasingly used in the frail and older population.

Survival from colorectal cancer is high for early stage disease but declines sharply with advanced stage at diagnosis. Table 1 shows the relationship between cancer stage and 5-year survival.

**Table 1:** Five-year relative survival by stage in England and Wales: adults aged 15–99; 2002–2006 Former Anglia Cancer Network (from Cancer Research UK)

Stage	Men (%)	Women (%)
Stage I	94.6	100.2
Stage II	83.5	85.9
Stage III	62.6	62.7
Stage IV	6.9	8.1
All stages	58.2	61.1
Stage not known	18.7	15.1

UK figures suggest that cancer survival rates are lower when compared to those in other Western European countries. Currently, the overall 5-year survival for colorectal cancer is around 50 % [3, 4]. It has been estimated that 10,000 deaths could be avoided each year if the UK cancer mortality figures were similar to the lowest rates in Europe [4]. Presently, the UK has only the 10th lowest male and 14th lowest female colorectal cancer-specific mortality rates in Europe [5]. The UK’s underperformance within Europe is felt to be in part related to a disproportionately high number of patients presenting at an advance stage or as an emergency. Approximately 25 % of patients with colorectal cancer present as an emergency, this proportion rises in the elderly and is higher than in many other common cancers [6]. This has a direct effect on 1-year survival rates; 48 % in those presenting as emergencies compared with 73 % through other routes of presentation [4].

### Two-week wait referral

**Fig. 1:**
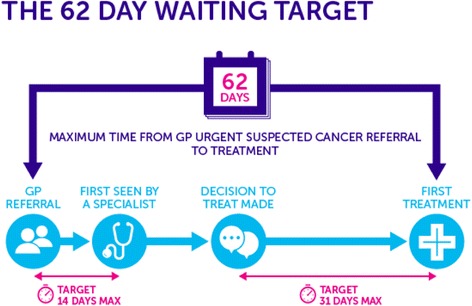
The 2-week urgent referral pathway (taken from Cancer Research UK website)

Following recognition by the Government and health bodies that patients were facing unacceptable waiting times for assessment, diagnosis and treatment of cancer, a new rapid access strategy was introduced through the Department of Health NHS Cancer plan in 2000. The 2-week wait (TWW) referral pathway was devised to streamline referral for those with symptoms suggestive of cancer in order to allow diagnosis at an earlier stage, reduce cancer survival inequality around the country and ultimately reduce cancer-related mortality [7]. By 2001, the Government pledged that those with suspected breast cancer would wait no more than 32 days from referral to diagnosis and this would extend to all cancer by 2005. Targets then focussed not only on diagnosis but treatment, meaning a wait no longer than 62 days from referral to cancer treatment (see Fig. 1). The Government plans to reduce the diagnostic timeframe from 32 to 28 days by 2020 [3].

Patients with symptoms suggestive of cancer predominantly present to primary care. But although cancer is common, in the primary care setting, it is still infrequently seen; a GP working in an average size practice will only see eight or nine new cancers per year, and only one colorectal cancer [8]. Thus in collaboration with The National Institute for Health and Care Excellence (NICE), a set of referral criteria to enable standardisation between practices were devised and rolled out alongside the Government referral targets for each suspected cancer site. Criteria include a combination of signs, symptoms and laboratory tests to be used in primary care and were updated in June 2015 [9]. Patients fulfilling this criteria are eligible to be seen within 14 days of referral.

Decisions regarding which symptoms would be included were based on a risk threshold, correlating to positive predictive value (PPV), i.e. if the risk of symptoms being caused by the specific cancer was above a pre-defined level, then further investigations are indicated [9]. The current NICE guidelines are based on PPV threshold of 3 % for symptoms being caused by cancer; this has decreased from the previous guideline. Recommendations are made based on the best available evidence, prediction models and collaborator expert opinion. Additional file 1 lists the referral criteria for suspected colorectal cancer with the new additions highlighted in brackets; in particular, there has been new emphasis placed on faecal occult blood testing [9]. According to the Department of Health, the original target was to ‘identify up to 90 % of patients with bowel cancer’ [7].

The system has been met with some controversy. A recent study gauging clinician opinion demonstrated significant concern over the disproportionate utilisation of services due to the TWW system, often to the detriment of patients who do not fit this criteria, but make up a greater proportion of referrals [10].

Currently, no referral target numbers or limitations have been set per population but at least 95 % of patients referred via this route must be seen within 14 days of referral. According to Department of Health figures, compliance is high with over 95 % of patients being seen within 2 weeks [3].

There are a number of drawbacks to a referral system based mainly on symptoms for colorectal cancer. Firstly, the criteria used for screening people visiting GPs with complaints/symptoms suggestive of colorectal cancer may lack predictive accuracy; many of those with colorectal cancer will not present with typical symptoms or indeed any symptoms at all. Studies suggest that only around half of patients diagnosed with colorectal cancer have symptoms that fit the 2-week wait criteria [8]. Of the total numbers diagnosed with colorectal cancer in England and Wales each year, only 27–30 % are diagnosed through the TWW route [11, 12]. Just 9 % of 2-week wait referrals result in diagnosis and treatment of colorectal cancer [10].

Secondly, the National Audit Office reported in 2010 a more than fourfold variation in referral rates across England and Wales. Although some of this can be explained by variations in proportions of the elderly and the impact of social differences such as smoking and alcohol intake, this does not fully explain the referral behaviour [13]. Poor adherence to TWW referral guidelines has been reported elsewhere too [14]. Work is underway to understand this variability further, but this suggests that many are either inappropriately referred or those with genuine symptoms are not being referred.

With the exception of survival measures, it is difficult to evaluate the effectiveness of a healthcare intervention or strategy aimed at cancer because definitions of what constitutes effective intervention are multi-faceted. Certainly, the measures of cost-effectiveness are complex when evaluating health interventions. But given the magnitude of the TWW policy and pressure it places on a stretched NHS resources, plus the Government plans to tighten the referral timeframes even further, it is vital that we explore and have an accurate understanding of its impact on health-related outcomes based on the available literature.

To the best of our knowledge, this evidence has not been systematically reviewed. In 2006, Thorne et al. reviewed and evaluated the effect of TWW on colorectal cancer and found low colorectal cancer detection rates with no improvement in stage at diagnosis compared to other referral routes (excluding emergency admissions) [15]. The poor reporting quality and limitations in methods of this review made it hard to replicate the study. For example, the report lacked a detailed search strategy, study inclusion criteria, study selection and extraction processes, quality assessment and the Preferred Reporting Items for Systematic Reviews and Meta-Analyses (PRISMA) study flow diagram [16].

Given the abovementioned gaps in evidence, there is a need for an updated systematic review assessing the effect of the 2-week wait system on the detection rate, stage at diagnosis and mortality from colorectal cancer in England and Wales.

### Objectives

The aim of this systematic review is to evaluate the effect of patient referral via the TWW system for suspected colorectal cancer on the detection rate, stage at diagnosis and mortality from colorectal cancer in England and Wales. The proposed systematic review will address the following questions:

- Primary outcomes
  - What is the proportion of patients investigated for suspected colorectal cancer via the England and Wales TWW system, who are later diagnosed with colorectal cancer (cancer conversion rate)?
  - What is the colorectal cancer stage at diagnosis?
  - What is the effect of the TWW system on the mortality due to colorectal cancer?
- Secondary outcomes
  - What is the proportion of patients diagnosed with a cancer outside of the colorectum who are referred via TWW with suspected colorectal cancer, and what site is affected?
  - What proportion of patients are meeting the TWW time targets (i.e. proportion of patients seen within 14 days of referral, diagnosed within 31 days of referral and started treatment within 62 days of referral)?

## Methods

This systematic review protocol is reported according to recommendations from the Preferred Reporting Items for Systematic Review and Meta-analysis Protocols (PRISMA-P) 2015 statement [17]; this is included as an Additional file 2.

### Study eligibility

All studies reporting the effects of TWW system for suspected colorectal cancer on the detection rate, stage at diagnosis and mortality from colorectal cancer will be eligible for inclusion.

- Inclusion criteria
  - Study design: Prospective or retrospective cohort studies, case-control studies, cross-sectional studies.
  - Study setting: Hospital- or community-based studies conducted in the UK.
  - Population: Patients 18 years or older with signs or symptoms suggestive of lower gastrointestinal cancer as dictated by the NICE guidance for cancer referral from primary care (NG12).
  - Intervention: TWW cancer referral system. Studies reporting data on multiple speciality referrals via TWW will be included if it is possible to separate outcome data by speciality to enable extraction of the colorectal cancer data only.
  - Comparator: Any comparator used by an individual study providing data on colorectal cancer detection, stage and/or mortality (e.g. referral via non-TWW system, ‘routine referral pathway’, first presentation of colorectal cancer via emergency admission and possibly the bowel cancer screening population). Studies with TWW data, but without a comparator arm will also be included.
  - Outcome: Rate of diagnosis, stage at diagnosis and/or mortality from colorectal cancer. Studies which include data on secondary outcomes without primary outcome data will be included.
  - Timing: Any length of follow-up.
  - Language of publication: English only.
  - Date of publication: Eligible studies published in the year 2000 or onwards to correspond with implementation of the TWW referral system.
  - Type of publication: Full-text reports.
- Exclusion criteria
  - Population: (a) Patients with suspected cancer other than colorectal, (b) anal cancer cases, (c) participants aged <18 years and (d) pregnant women.
  - Intervention/comparator: Non-TWW referral system (e.g. urgent referral, routine referral only). Non-UK-based studies.
  - Outcomes: Studies not including data on any of the three primary outcomes (colorectal cancer diagnosis, stage and/or mortality) or secondary outcomes.
  - Publication type: Reviews, editorials, letters, books, consensus statements or opinions. Review articles will be examined for identification of original studies but will not be included if they contain no primary data.

### Search strategy

The specific search strategies will be employed by a librarian with experience in systematic review searches; input will then be obtained from the review team. We will search Cochrane, EMBASE, MEDLINE via PubMed, NHS Evidence, Trip and the British Library Catalogue using a combination of subject headings and keywords related to the 2-week wait referral pathway and colorectal cancer and outcomes. Only UK-conducted studies will be included. We will employ a series of search strategies using increasingly refined search terms to increase chances of including all relevant publications, as some may focus on TWW clinic outcomes for a range of specialities. Where multi-speciality studies occur, the full publication will be reviewed and if data can be extrapolated for colorectal cancer in isolation, it will be included in the review. The search criteria will be peer reviewed by RA and NW both with systematic review experience.

In addition, we will hand review the reference list of possible eligible articles, letters, as well as reviews and search Google Scholar for any potential additional articles not included in the above searches, such as PhD theses and government reports. We will perform individualised searches on authors of included studies. Conference work will not be searched for or included as they do not represent full text and therefore would not allow sufficient information to verify our desired outcome data. We do not anticipate relevant publications prior to 2000 as the TWW system was implemented that year; therefore, searches will be limited to 2000 and onwards. PROSPERO will be searched to ensure no ongoing systematic review on the subject matter is already in place. We will update the search before completion of the review.

The search strategy is documented in detail in Additional file 3.

### Data management and study selection

All publications will be collated and de-duplicated then entered into a specialised database using a Clinical Evidence Based Information Service (CEBIS). Four reviewers (EM, MM, MT, MW) will use a screening form defining eligibility criteria to independently screen all titles and abstracts. Full texts of all potentially eligible records passing the abstract/title screening level will be retrieved and reviewed for their inclusion in the review by two pairs of reviewers (EM and MM plus MT and MW). At both screening levels, all disagreements between the reviewers will be discussed and resolved through discussions or a third party adjudication. Further information will be gathered from authors where questions regarding eligibility arise. Reasons for trial exclusion will be documented and presented in the PRISMA study flow diagram.

### Data extraction

Using a standardised and pre-piloted data extraction form, four reviewers (EM, MM, MW, MT) will ascertain and extract the following relevant information from included publications: study (authors, publication year and country, design, setting, sample size and study follow-up duration), patient characteristics (e.g. demographics, co-morbidities) and primary/secondary outcome measures. Any disagreements will be resolved by consensus discussion plus involvement of either RA or NW. Missing data will either be calculated, provided the necessary data is available, or the authors will be contacted, if possible.

### Primary outcomes

- The rate of colorectal cancer diagnosis in patients referred through the TWW system
- Colorectal cancer stage at diagnosis (the staging system used will be determined by each individual study as it is anticipated the system used will vary between studies)
- Colorectal cancer related mortality amongst subjects referred and diagnosed via TWW

### Secondary outcomes

- Other diagnosis made via the 2-week wait referral system except colorectal cancer
- Adherence to referral time frame protocol (e.g. seen by specialist within 14 days of referral)

Data extraction sheet is provided in Additional file 4: Table S1.

### Risk of bias assessment

Risk of bias will be assessed by two pairs of reviewers (EM and MM and MW and MT) using the Scottish Intercollegiate Guidelines Network (SIGN) checklists for cohort and case-control studies [18, 19]. These validated tools address several domains of bias (study research question, participant selection, information bias, confounding, statistical analysis and study’s summary judgement on internal and external validity). The overall quality ratings (high, acceptable or low) will be based on the extent to which the pre-selected domains will be affected in cohort (items 4–5, 7, 10–11 and 13) and case-control studies (items 3–4, 6–7, 9 and 10). We anticipate a significant proportion of the studies to be of low quality; therefore, we will look first at high/acceptable quality studies but where appropriate, we will carry out a sensitivity analysis including low-quality studies, or if the data does not permit, we will describe the low-quality studies in the discussion. The quality assessments will be cross-checked, and any disagreement will be resolved via group discussion. Both quality assessment tools are provided in Additional file 5: Tables S2 and S3.

### Data analysis and synthesis

The characteristics and findings of the included studies will be narratively synthesised. Evidence will be organised into tables and text to denote the aggregated information on study type and participants. The synthesised data will be presented separately for each primary outcome using tables and figures. For each comparative study, the dichotomous summary effect measures for the post-referral colorectal cancer diagnosis and mortality rates will be calculated and expressed as relative risk (RR), hazard rate ratio (HRR) or risk difference (RD) with corresponding 95 % confidence intervals (95 % CIs). The post-referral cancer stage at diagnosis will be treated as either dichotomous or continuous outcome measure. As the continuous measure, it will be expressed as mean difference (MD) with 95 % CIs. The summary effect measures will be pooled across studies, only if there is sufficient similarity in study design, settings, participants (age, sex, comorbidity), comparators (e.g. similar referral systems), length of follow-up and primary outcome measures (e.g. colorectal cancer diagnostic criteria, staging system and scale of measurement). The pooled estimates of RR and MD with corresponding 95 % CIs will be generated using the random-effects meta-analysis model by DerSimonian and Laird [20]. The heterogeneity and its extent for the pooled estimates will be assessed via visual inspection of forest plots and statistical test results (chi-square <0.10 and the *I* ^2^ statistic >50 %).

If data permits, we will conduct a subgroup analysis to investigate if any of the a priori selected factors (age, symptoms at presentation, colorectal cancer site and stage) explain the observed heterogeneity by modifying the effects of TWW referral system on the primary outcomes.

Given the sufficient number of data points, publication bias will be assessed by inspecting funnel plot asymmetry and using linear regression tests [21].

## Discussion

In this systematic review, we aim to summarise the relevant evidence on cancer detection rate, cancer stage at diagnosis and disease-related mortality rates for patients with suspected colorectal cancer going through the TWW referral system in England and Wales. We will also, where data allows, present details of other diagnosis made on the same group of patients plus data on adherence to the timeframes of the TWW process. We will, where possible, highlight gaps in the evidence. We aim to provide a better understanding of the TWW referral system in England and Wales for the investigation of colorectal cancer, in particular whether it is meeting its desired effect to diagnose the majority of colorectal cancers and potentially by diagnosing more at an earlier stage reducing colorectal cancer related deaths.

## Additional files

Additional file 1: NICE primary care referral guidelines for suspected cancer. (PDF 339 kb)
Additional file 2: PRISMA-P checklist, word document. This is the populated PRISMA-P checklist. (DOC 81 kb)
Additional file 3: Search strategies for PubMed MEDLINE. (PDF 806 kb)
Additional file 4: Table S1. Data extraction sheet sample. (PDF 998 kb)
Additional file 5: Risk of bias assessment of included studies. **Table S2.** Risk of bias in cohort studies [16 items]. **Table S3.** Risk of bias in case-control studies [13 items]. (PDF 1105 kb)

## Abbreviations

CEBIS: Clinical Evidence Based Information Service
CI: Confidence interval
GP: General Practitioner
HRR: Hazard rate ratio
MD: Mean difference
NHS: National Health Service
NICE: National Institute for Health and Care Excellence
PPV: Positive predictive value
PRISMA-P: Preferred Reporting Items for Systematic Review and Meta-analysis Protocols
RD: Risk difference
RR: Relative risk
SIGN: Scottish Intercollegiate Guidelines Network
