# Supplementary material for: The effect of the 2-week wait referral system on the detection of and mortality from colorectal cancer: protocol of a systematic review and meta-analysis
Source: Syst Rev. 2016 Oct 26;5:182. doi: 10.1186/s13643-016-0358-6 (PMC5081696; doi:10.1186/s13643-016-0358-6)
Supplement: Additional file 1: — NICE primary care referral guidelines for suspected cancer. (PDF 339 kb) [file 13643_2016_358_MOESM1_ESM.pdf]

Additional file 1

NICE Primary care referral guidelines for suspected cancer

1.3 Lower gastrointestinal tract cancers

Colorectal cancer

1.3.1 Refer people using a suspected cancer pathway referral [for an appointment within 2 weeks] for colorectal cancer if:

- they are aged 40 and over with unexplained weight loss and abdominal pain **or**
- they are aged 50 and over with unexplained rectal bleeding **or**
- they are aged 60 and over with:
  - iron-deficiency anaemia **or**
  - changes in their bowel habit, **or**
- tests show occult blood in their faeces [see recommendation 1.3.4 for who should be offered a test for occult blood in faeces]. **[new 2015]**

1.3.2 Consider a suspected cancer pathway referral [for an appointment within 2 weeks] for colorectal cancer in people with a rectal or abdominal mass. **[new 2015]**

1.3.3 Consider a suspected cancer pathway referral [for an appointment within 2 weeks] for colorectal cancer in adults aged under 50 with rectal bleeding **and** any of the following unexplained symptoms or findings:

- abdominal pain
- change in bowel habit
- weight loss
- iron-deficiency anaemia. **[new 2015]**

1.3.4 Offer testing for occult blood in faeces to assess for colorectal cancer in adults without rectal bleeding who:

- are aged 50 and over with unexplained:
    - abdominal pain **or**
    - weight loss, **or**
- are aged under 60 with:
    - changes in their bowel habit **or**
    - iron-deficiency anaemia, **or**
- are aged 60 and over and have anaemia even in the absence of iron deficiency. **[new 2015]**
