# Supplementary material for: The effect of the 2-week wait referral system on the detection of and mortality from colorectal cancer: protocol of a systematic review and meta-analysis
Source: Syst Rev. 2016 Oct 26;5:182. doi: 10.1186/s13643-016-0358-6 (PMC5081696; doi:10.1186/s13643-016-0358-6)
Supplement: Additional file 3: — Search strategies for PubMed MEDLINE. (PDF 806 kb) [file 13643_2016_358_MOESM3_ESM.pdf]

Additional file 3

Search strategies for Pubmed MEDLINE

1. **[[["Referral and Consultation"[Mesh]]] AND "Neoplasms"[Mesh]] AND "Waiting Lists"[Mesh]**
2. **[[[[fast track OR rapid OR urgent]]] AND ["Referral and Consultation"[Mesh]]] AND "Neoplasms"[Mesh]**
3. **[[[[[[outcomes OR survival OR diagnosis OR detection]]] AND [[gastrointestinal OR colorectal OR oesophageal OR stomach OR duodenal OR bowel OR rectal OR CRC]]] AND [[TWW OR 2WW OR two week wait OR rapid access OR fast track OR urgent referral]]]] AND cancer**
