# Supplementary material for: The effect of the 2-week wait referral system on the detection of and mortality from colorectal cancer: protocol of a systematic review and meta-analysis
Source: Syst Rev. 2016 Oct 26;5:182. doi: 10.1186/s13643-016-0358-6 (PMC5081696; doi:10.1186/s13643-016-0358-6)
Supplement: Additional file 4: Table S1. — Data extraction sheet sample. (PDF 998 kb) [file 13643_2016_358_MOESM4_ESM.pdf]

Additional file 4

Table S1. Data extraction sheet sample

**Study details**
**First author surname year of publication**:<br><br>**Country**:<br><br>**Study design**:<br><br>**Study setting** [primary care/specialty clinic/other - specify]:<br><br>**Number of centres**:<br><br>**Total length of follow up**:<br><br>**Funding** [government/private/manufacturer/other - specify]:
**Aim of the study**
**Participants**
**Recruitment dates**:<br><br>**Sample size:**<br><br>**Inclusion criteria**:<br><br>**Exclusion criteria**:<br><br><br>**Characteristics of participants [total study sample]**<br><br>Mean [range or SD] age [years]:<br><br>Women [n [%]]:<br><br>Race/ethnicity [n [%]]:
**Intervention**
**TWW referral group:**<br><br>**Intervention 2 group**:<br><br>**Intervention 3 group**:
**Outcomes [study-based]**

**Primary outcomes** [*list*]:<br><br><br>**Secondary outcomes** [*list*]:

Number of patients

	**TWW group**	**Intervention 2 group**	**Intervention 3 group**
**Referred for further investigations**			
**Analysed**<br>[If more than one follow-up, choose and specify the last one]			
**Losses to follow-up/drop out/sample attrition**<br>[If more than one follow-up, choose and specify the last one]			

Interventions

	**Description**
TWW referral group	
Intervention 2 group	
Intervention 3 group	

Patient baseline characteristics

	**TWW referral group**	**Intervention 2 group**	**Intervention 3 group**
**Age [years]**<br>Mean [SD]			
**Sex –female** n/N [%]			
**BMI** [kg/m^2]<br>Mean [SD]			
**Smoking** n/N [%]			

Efficacy outcomes

	TWW referral group	Intervention 2 group	Intervention 3 group	Between-group difference p value [or 95% CI]*
**Primary Outcomes**				
Diagnosed with CRC % [n/N]				
CRC stage at diagnosis				
Mortality from CRC				
**Secondary outcomes**				
Other diagnosis made				
Adherence to diagnostic/treatment targets				
**Authors conclusion**				
**Reviewer's conclusion**				
