# Supplementary material for: The effect of the 2-week wait referral system on the detection of and mortality from colorectal cancer: protocol of a systematic review and meta-analysis
Source: Syst Rev. 2016 Oct 26;5:182. doi: 10.1186/s13643-016-0358-6 (PMC5081696; doi:10.1186/s13643-016-0358-6)
Supplement: Additional file 5: — Risk of bias assessment of included studies. Table S2. Risk of bias in cohort studies [16 items]. Table S3. Risk of bias in case-control studies [13 items]. (PDF 1105 kb) [file 13643_2016_358_MOESM5_ESM.pdf]

Additional file 5

Risk of bias assessment of included studies

Table S2. Risk of bias in cohort studies [16 items]

Definition [Item #]	Study 1	Study 2	Study 3	Study 4	Study 5
**Internal validity**					
The study addresses an appropriate and clearly focused question [Item 1]					
**Selection of subjects**					
The two groups being studied are selected from source populations that are comparable in all respects other than the factor under investigation [Item 2]					
The study indicates how many of the people asked to take part did so, in each of the groups being studied [Item 3]					
The likelihood that some eligible subjects might have the outcome at the time of enrolment is assessed and taken into account in the analysis [Item 4]					
What percentage of individuals or clusters recruited into each arm of the study dropped out before the study was completed [Item 5]					

Comparison is made between full participants and those lost to follow up, by exposure status [Item 6]					
**Assessment**					
The outcomes are clearly defined [Item 7]					
The assessment of outcome is made blind to exposure status. If the study is retrospective this may not be applicable [Item 8]					
Where blinding was not possible, there is some recognition that knowledge of exposure status could have influenced the assessment of outcome [Item 9]					
The method of assessment of exposure is reliable [Item 10]					
Evidence from other sources is used to demonstrate that the method of outcome assessment is valid and reliable [Item 11]					
Exposure level or prognostic factor is assessed more than once [Item 12]					
**Confounding**					
The main potential confounders are identified and taken into account in the design and analysis [Item 13]					
**Statistical analysis**					
Have confidence intervals been provided? [Item 14]					
**Overall assessment of the study**					
Taking into account clinical considerations, your evaluation of the methodology used, and the statistical					

power of the study, do you think there is clear evidence of an association between exposure and outcome?<br><br>[Item 15]					
Are the results of this study directly applicable to the patient group targeted in this guideline? [Item 16]					
**Summary quality [risk of bias] rating**					

NA=not applicable

Possible responses to each item: yes, no, can't say, or doesn't apply

High quality^{++} [little or no risk of bias; results unlikely to be changed by further research]

Acceptable quality^{+} [most criteria met; some flaws in the study with an associated risk of bias; conclusions may change in the light of further studies]

Low quality ^{0} [either most criteria not met, or significant flaws relating to key aspects of study design; conclusions likely to change in the light of further studies]

The overall methodological quality of each study are based on the extent to which the pre-selected important domains of bias were affected [response 'no' or 'can't say'].

For cohort studies, these items were the following by each domain of bias:

- Selection of subjects [items 4-5]
- Assessment [item 7, items 10-11]
- Confounding [item 13]

Table S3. Risk of bias in case-control studies [13 items]

Definition [Item #]	Study 1	Study 2	Study 3	Study 4	Study 5
**Internal validity**					
The study addresses an appropriate and clearly focused question [Item 1]					
**Selection of subjects**					
The cases and controls are taken from comparable populations [Item 2]					
The same exclusion criteria are used for both cases and controls [Item 3]					
What percentage of each group [cases and controls] participated in the study? [Item 4]					
Comparison is made between participants and non-participants to establish their similarities or differences [Item 5]					
Cases are clearly defined and differentiated from controls [Item 6]					
It is clearly established that controls are non-cases [Item 7]					
**Assessment**					
Measures will have been taken to prevent knowledge of primary exposure influencing case ascertainment [Item 8]					
Exposure status is measured in a standard, valid and reliable way [Item 9]					
**Confounding**					

The main potential confounders are identified and taken into account in the design and analysis [Item 10]					
**Statistical analysis**					
Confidence intervals are provided [Item 11]					
**Overall assessment of the study**					
Taking into account clinical considerations, your evaluation of the methodology used, and the statistical power of the study, do you think there is clear evidence of an association between exposure and outcome? [Item 12]					
Are the results of this study directly applicable to the patient group targeted by this guideline? [Item 13]					
**Summary quality [risk of bias] rating**					

Possible responses to each item: yes, no, can't say, or doesn't apply

High quality++ [little or no risk of bias; results unlikely to be changed by further research]

Acceptable quality+ [most criteria met; some flaws in the study with an associated risk of bias; conclusions may change in the light of further studies]

Low quality 0 [either most criteria not met, or significant flaws relating to key aspects of study design; conclusions likely to change in the light of further studies]

The overall methodological quality of each study are based on the extent to which the pre-selected important domains of bias were affected [response 'no' or 'can't say'].

For case-control studies, these items were the following by each domain of bias:

- Selection of subjects [items 3-4, items 6-7]
- Assessment [item 9]

- Confounding [item 10]
